# The Emerging Role of Transcription Factor Spi-C in Macrophage Biology and Inflammatory Pathogenesis

**DOI:** 10.3390/ijms27041730

**Published:** 2026-02-11

**Authors:** Md Zahidul Alam, Weihua Huang

**Affiliations:** Department of Pathology and Laboratory Medicine, Brody School of Medicine, East Carolina University, 600 Moye Boulevard, Greenville, NC 27834, USA

**Keywords:** iron recycling macrophages, ETS family of transcription factors, toll-like receptors, transcription factor Spi-C, inflammation, ferroptosis

## Abstract

Spi-C is a member of the ETS (E26 transformation-specific) family of transcription factors, a group of proteins that regulate gene expression in animals by binding to specific DNA sequences. Spi-C has emerged as a central regulator of macrophage adaptation to iron exposure, inflammatory stress, and tissue injury. Studies show that Spi-C programs iron-recycling macrophages by promoting expression of key iron-handling genes, thereby supporting iron efflux, safe intracellular iron storage, and the development of red pulp macrophages critical for systemic iron recycling. Its expression is strongly induced by heme and iron, enabling macrophages to respond adaptively to increased heme turnover, whereas Spi-C deficiency leads to impaired iron recycling and pathological iron accumulation. Beyond iron homeostasis, Spi-C is increasingly recognized as a regulator of inflammatory disease, functioning as an anti-inflammatory and tissue-protective factor across multiple models, including lipopolysaccharide (LPS)–induced systemic inflammation and colitis, where Spi-C deficiency leads to enhanced cytokine production, increased tissue injury, and impaired repair. By integrating NF-κB-driven inflammatory cues with metabolic adaptation, Spi-C maintains macrophage homeostasis across tissues. This short review summarizes these known functions and provides a forward-looking perspective that Spi-C may also regulate macrophage susceptibility to ferroptosis, an iron-dependent form of cell death implicated in diverse inflammatory and degenerative conditions.

## 1. Introduction: Spi-C in the ETS Transcription Factor Network

The ETS (E26 transformation-specific) family of transcription factors plays a central role in hematopoietic development and immune cell function by translating extracellular signals into lineage-specific transcriptional programs [1,2,3,4]. Among immune cells, ETS factors are particularly critical in myeloid and lymphoid lineages, where they regulate cell fate decisions, activation states, and functional specialization [1]. Canonical members such as PU.1 and Spi-B are well-established master regulators of myeloid and B cell differentiation, respectively, functioning through extensive genome-wide occupancy at promoters and enhancers that define immune cell identity [5,6,7,8]. In contrast, Spi-C (*Spic*), a closely related ETS family member, remained comparatively understudied for many years, largely due to its restricted expression pattern and the absence of overt developmental defects in early hematopoiesis [9,10].

Initial studies characterized Spi-C as a PU.1-related factor expressed at low levels in select B cell subsets, leading to the prevailing view that Spi-C functioned as a niche-restricted or redundant transcription factor with limited physiological relevance [10]. Unlike PU.1, which is broadly expressed across monocytes, macrophages, dendritic cells, and B cells, Spi-C appeared dispensable for general immune development, reinforcing the perception that it played only a minor or context-specific role within the ETS transcriptional network. This view began to shift with the discovery that Spi-C is indispensable for the development of splenic red pulp macrophages, revealing for the first time that Spi-C functions as a lineage-defining transcription factor rather than a passive modifier of existing programs [11].

Subsequent work has dramatically expanded this narrow perspective. Spi-C is now recognized as a key regulator of macrophage adaptation to iron-rich and inflammatory environments, where it integrates metabolic cues—particularly heme and iron—with transcriptional control of erythrophagocytosis, iron recycling, and inflammatory tone. Beyond splenic red pulp macrophages, Spi-C expression and function have been identified in erythroblastic island macrophages, intestinal macrophages, and activated macrophages responding to Toll-like receptor signaling [12,13,14]. In parallel, emerging studies demonstrate that Spi-C plays non-redundant roles in B cell fate decisions and in embryonic stem cells, where it links cellular metabolism to epigenetic regulation [10,15]. Together, these findings establish Spi-C as a transcription factor with broad relevance across immune and non-immune systems, far exceeding its originally perceived niche.

A defining feature of Spi-C biology is its regulation by environmental and metabolic signals rather than by classical developmental cues alone. Heme-mediated relief of BACH-dependent repression, NF-κB-driven induction during inflammation, and counter-regulation by interferon signaling position Spi-C as a transcriptional sensor that responds dynamically to tissue context [14,16]. Through this regulatory architecture, Spi-C enables immune cells to adapt their identity and function in response to metabolic stress, iron availability, and inflammatory burden, rather than enforcing a fixed differentiation state.

Given Spi-C’s central role in macrophage iron handling, it is relevant to consider how iron-driven oxidative processes influence macrophage fate. Iron-catalyzed lipid peroxidation has emerged as a key driver of inflammatory tissue injury, providing a mechanistic bridge between iron metabolism and regulated cell death pathways such as ferroptosis [17,18].

**Figure 1:**
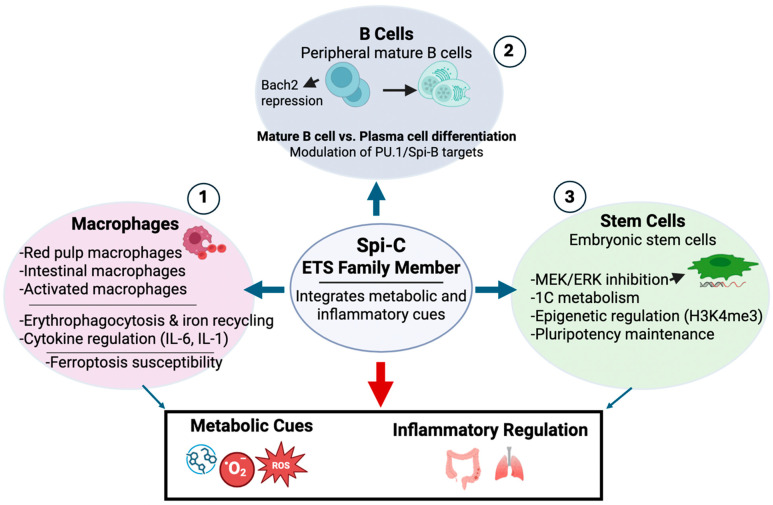
Spi-C integrates metabolic and inflammatory cues to regulate immune and stem cell programs. Spi-C, an ETS family transcription factor, functions as a context-dependent regulator linking metabolic stress and inflammatory signaling across multiple cell types. (**1**) In macrophages, Spi-C controls erythrophagocytosis and iron recycling, modulates inflammatory cytokine expression, and influences susceptibility to ferroptotic stress. (**2**) In peripheral B cells, Spi-C shapes mature B cell versus plasma cell differentiation through repression of Bach2 and modulation of PU.1/Spi-B target genes. (**3**) In embryonic stem cells, Spi-C contributes to pluripotency maintenance via metabolic, signaling, and epigenetic mechanisms. Together, Spi-C acts as a transcriptional node coordinating metabolic cues with inflammatory regulation across tissues. Created in BioRender. Alam, Z. (2026) https://BioRender.com/04yfm9v.

In this review, we discuss current knowledge of Spi-C function across macrophages, B cells, and stem cells, with particular emphasis on its emerging role as an integrator of metabolic and inflammatory signals. We propose a unifying framework in which Spi-C operates not simply as a lineage marker, but as a context-dependent transcriptional switch that shapes immune cell identity, inflammatory balance, and metabolic adaptation (Figure 1). Because Spi-C sits at the intersection of iron handling, redox stress, and inflammatory signaling, its activity may also influence how immune cells respond to iron-dependent oxidative damage, linking transcriptional regulation to broader pathways of cellular stress and survival. Understanding how Spi-C functions within the broader ETS transcription factor network will therefore be critical for deciphering immune cell plasticity and vulnerability in health and disease.

## 2. Discovery of Spi-C as a Lineage-Defining Factor in Red Pulp Macrophages

### 2.1. Spi-C and Red Pulp Macrophage Development

The transcription factor Spi-C was first identified as a PU.1-related ETS family member with reported expression in B cells; however, its physiological function remained unclear for nearly a decade. This changed with the landmark study by Kohyama and colleagues, which revealed Spi-C as a lineage-defining transcription factor selectively required for splenic red pulp macrophage (RPM) development [11]. Among hematopoietic populations, Spi-C expression was found to be highly enriched in RPMs, while remaining low or undetectable in circulating monocytes, dendritic cells, and other tissue-resident macrophage subsets [11]. This restricted expression pattern immediately distinguished Spi-C from broadly acting myeloid transcription factors.

Genetic ablation of *Spic* resulted in a near-complete and selective loss of RPMs, without perturbing monocyte development, other macrophage populations, or lymphoid lineages. Importantly, mixed bone marrow chimera and retroviral rescue experiments demonstrated that this defect was cell-autonomous, arising from intrinsic failure of Spi-C–deficient hematopoietic cells to generate RPMs [11]. Restoration of Spi-C expression in Spic^-^/^-^ bone marrow fully rescued RPM development, establishing Spi-C as both necessary and sufficient for this lineage. These findings demonstrated that RPMs are not merely terminally differentiated macrophages shaped by environmental cues but instead represent a distinct macrophage lineage whose identity requires Spi-C-dependent transcriptional programming. These observations positioned Spi-C as one of the first transcription factors shown to selectively govern the development of a specialized tissue-resident macrophage subset.

However, beyond cell-intrinsic defects, it is plausible that loss of Spi-C-dependent red pulp macrophages could also perturb the splenic microenvironment. Impaired erythrocyte clearance and disrupted iron recycling may promote local iron accumulation and oxidative stress, while altered macrophage interactions with stromal or endothelial cells could reshape niche-derived signals. Although these non–cell-autonomous effects have not yet been directly examined, they represent a potential mechanism by which Spi-C deficiency might influence other immune and hematopoietic populations within the splenic niche.

### 2.2. Spi-C in Erythrocyte Clearance and Iron Homeostasis

Beyond its role in RPM development, Spi-C was shown to be essential for the core physiological function of RPMs—erythrocyte clearance and iron recycling. RPMs are strategically located within the splenic red pulp, where they phagocytose senescent or damaged red blood cells (RBCs), liberate heme-derived iron, and return it to systemic circulation [19,20,21]. In Spic-deficient mice, circulating RBCs were efficiently trapped within the spleen, indicating preserved mechanical filtration; however, phagocytosis of these RBCs was profoundly impaired [11]. This defect correlated directly with the absence of F4/80^hi^ CD68^+^ RPMs, identifying Spi-C-dependent macrophages as the dominant erythrophagocytic population in the spleen.

Failure of RBC clearance had striking metabolic consequences. Spic^-^/^-^ mice developed progressive splenomegaly and a marked accumulation of iron localized specifically to the splenic red pulp, while liver and serum iron levels remained largely normal [11]. Histological and biochemical analyses confirmed that iron overload was not systemic but instead reflected defective iron handling within the splenic niche. Importantly, erythropoiesis and circulating erythrocyte parameters were unaltered, indicating that the phenotype arose from impaired macrophage-mediated recycling rather than abnormal RBC production or survival.

At the molecular level, RPMs were shown to express high levels of genes involved in hemoglobin scavenging and iron transport, including Cd163 and Slc40a1 (ferroportin), as well as the vascular cell adhesion molecule VCAM-1, which was later identified as a direct Spi-C transcriptional target [11]. Loss of Spi-C resulted in coordinated downregulation of these programs, reinforcing the concept that Spi-C controls a functional gene network dedicated to iron and heme metabolism, rather than serving solely as a developmental switch. Although Spi-C is widely described as a lineage-defining factor for red pulp macrophages, its function likely encompasses both direct transcriptional specification and indirect niche-mediated effects. Spi-C promotes expression of genes required for erythrophagocytosis and iron recycling, and disruption of these programs may secondarily alter the splenic microenvironment, further destabilizing red pulp macrophage identity and maintenance.

Physiological erythrocyte turnover provides controlled, localized heme exposure that supports homeostatic Spi-C induction in iron-recycling macrophages. Under these conditions, heme functions primarily as a differentiation and maintenance signal within the splenic red pulp niche. Pathological hemolysis or tissue injury, however, can release excessive extracellular heme, leading to heightened oxidative stress and inflammatory signaling. Such conditions may qualitatively alter Spi-C regulation, shifting macrophages from a homeostatic iron-recycling program toward stress-responsive or pro-inflammatory states. Defining how these distinct heme environments differentially influence Spi-C-dependent transcriptional programs will be important for understanding macrophage adaptation in hemolytic and inflammatory diseases.

## 3. Heme as a Metabolite Signal: Induction and Regulation of Spi-C

The discovery of Spi-C as a lineage-defining transcription factor for red pulp macrophages raised a fundamental question: what upstream signals induce Spi-C expression in macrophages? Subsequent work identified heme—a metabolic byproduct of erythrocyte turnover—as the dominant physiological cue that drives Spi-C expression, establishing one of the clearest examples of metabolite-instructed immune cell differentiation.

### 3.1. Heme-Driven Differentiation Programs

Heme was identified as a potent and selective inducer of Spic expression in macrophages. Heme is an iron-containing prosthetic group released during macrophage-mediated clearance of senescent erythrocytes. Following erythrophagocytosis, heme is catabolized by heme oxygenase-1, liberating iron for recycling via ferroportin and limiting toxicity [22,23]. Exposure of bone marrow-derived macrophages (BMDMs) to heme robustly induced Spi-C, whereas classical inflammatory stimuli alone were insufficient to fully recapitulate this response [16]. Importantly, heme-induced Spi-C expression was not restricted to splenic red pulp macrophages, but extended to macrophages in other erythrocyte-processing niches, including erythroblastic island macrophages (EBIMφs) in the bone marrow [13]. EBIMφs are specialized bone marrow macrophages that support terminal erythroid differentiation by providing trophic signals, clearing extruded nuclei, and regulating local iron availability [24,25,26]. These findings revealed that Spi-C is not exclusively a splenic factor, but rather defines a conserved transcriptional program activated in macrophages exposed to high heme flux.

Heme-induced Spi-C expression was shown to be instructive rather than permissive, actively reprogramming macrophages toward an iron-recycling phenotype [16]. Induction of Spi-C correlated with upregulation of genes involved in hemoglobin uptake, iron export, and heme detoxification, reinforcing the concept that heme functions as a contextual metabolite signal that shapes macrophage identity to match local metabolic demands [16]. This paradigm challenged earlier views that macrophage specialization arises primarily from cytokine or pattern-recognition receptor signaling, positioning metabolic cues as central regulators of immune cell fate.

### 3.2. BACH1 and BACH2 as Transcriptional Gatekeepers of Spi-C

Mechanistic insight into heme-mediated Spi-C induction emerged with the identification of the BACH family of transcriptional repressors as direct upstream regulators of Spic. The BACH family of transcriptional repressors (primarily BACH1 and BACH2) are heme-sensitive transcription factors that bind Maf recognition elements (MAREs) to suppress target gene expression [27,28,29]. Under homeostatic conditions, BACH1 binds to Maf recognition elements within the Spic locus and actively represses its transcription. Heme binding to BACH1 triggers conformational changes that promote nuclear export, ubiquitination, and proteasomal degradation of BACH1, thereby relieving repression of Spic and permitting Spi-C expression [16]. Haldar et al. demonstrated that heme-induced Spi-C expression drives the differentiation of monocytes into iron-recycling macrophages, including F4/80^+^VCAM1^+^ bone marrow macrophages [16]. Under conditions of elevated heme, such as pathologic hemolysis, Spi-C is induced in monocytes through BACH1 degradation, enabling the generation of new iron-recycling macrophages despite transient depletion of existing RPMs or bone marrow macrophages. Mechanistically, cysteine-proline motifs within BACH1 are required for its heme-dependent degradation and for derepression of Spic [16].

Heme-driven Spi-C induction reflects the integration of multiple upstream signals that modulate transcriptional accessibility and macrophage phenotype. In bone marrow-derived macrophages (BMDMs), Haldar et al. found that heme triggers polyubiquitination and proteasome-dependent degradation of BACH1, a repressor of Spi-C [16]. Consistent with this, they observed that increasing heme levels reduced BACH1 protein while generating a higher molecular weight species, indicative of post-translational modification, likely polyubiquitination. Furthermore, proteasome inhibition blocked heme-mediated induction of Spic-EGFP^+^ BMDMs, supporting a role for ubiquitination and BACH1 degradation in Spi-C activation. In intestinal macrophages, heme inhibits a subset of LPS-induced pro-inflammatory genes, including Il6 and Il1a, by preventing formation of the IRF5–NF-κB p65 complex, thereby promoting a non-inflammatory phenotype, as will be discussed in more detail later in this review. Together, these findings indicate that heme relieves BACH-mediated transcriptional repression at Maf recognition elements within the Spic locus, while additional inputs from oxidative stress and inflammatory signaling further modulate the magnitude and tissue specificity of Spi-C induction. The relative contribution of these pathways likely varies between tissues, reflecting differences in erythrophagocytosis, redox environment, and cytokine exposure.

This heme–BACH1–Spi-C axis provides a direct molecular link between intracellular heme levels and transcriptional reprogramming. Notably, this regulatory logic extends beyond macrophages. In B cells, BACH2 serves an analogous repressive function at the Spic locus, highlighting cell-type–specific deployment of BACH family members to constrain Spi-C expression until appropriate metabolic cues are encountered [30]. These findings place Spi-C within a broader regulatory framework in which environmental metabolites directly control transcription factor availability, bypassing classical cytokine-driven signaling cascades.

The exquisite sensitivity of BACH proteins to heme underscores why Spi-C expression is tightly restricted under steady-state conditions and rapidly induced in iron-rich environments. This regulatory architecture ensures that Spi-C-dependent programs are activated only when macrophages encounter sustained heme exposure, thereby minimizing inappropriate iron accumulation or oxidative stress.

BACH1 and BACH2 function not only as basal repressors of Spi-C but also as dynamically regulated sensors during inflammation and tissue repair [31,32,33]. Inflammatory signaling, oxidative stress, and fluctuations in intracellular heme availability can alter BACH protein stability, post-translational modification, and nuclear occupancy, thereby reshaping repression at the Spic locus. As tissues transition from inflammatory injury toward resolution and remodeling, these context-dependent changes in BACH activity may permit reactivation of Spi-C-dependent transcriptional programs that support restoration of macrophage homeostasis and niche-specific functions.

### 3.3. Spi-C in the Erythroblastic Island Niche

Beyond its established role in splenic red pulp macrophages, Spi-C is a defining transcriptional regulator of erythroblastic island macrophages (EBIMφs), the specialized macrophages that form the core of erythroblastic islands and coordinate terminal erythropoiesis. EBIMφs physically associate with erythroblasts, providing trophic signals, mediating iron recycling, and clearing extruded nuclei [24]. Similarly to red pulp macrophages, EBIMφs express Spi-C in a heme-dependent manner and share a conserved transcriptional program enriched for iron-handling, adhesion, and erythroid-supportive genes [13].

Recent work by Noel et al. establishes Spi-C as a robust and functionally relevant marker of EBIMφ identity in vivo. Using Spi-C GFP reporter mice, the authors demonstrated that Spi-C expression distinguishes EBIMφs from other myeloid populations in the bone marrow, with Spi-C^+^ macrophages exhibiting elevated F4/80 and CD163 expression [13]. Further, Spi-C–GFP marked more than 90% of EBIMφs, supporting its utility as a lineage-defining transcription factor within the erythroblastic island niche. Mechanistically, Spi-C expression in EBIMφs is highly sensitive to systemic inflammatory cues. Noel et al. showed that burn injury induces granulocyte colony-stimulating factor (G-CSF) secretion, which broadly suppresses EBIMφ marker genes, including *Spic* and *Cd163*, and leads to a quantitative loss of Spi-C^+^ EBIMφs in the bone marrow [13]. This reduction was accompanied by diminished erythroid cell populations and impaired erythropoiesis, and G-CSF administration alone was sufficient to phenocopy these effects.

These findings position Spi-C as a transcriptional node that integrates heme-derived metabolic signals with inflammatory cytokine inputs to maintain erythroblastic island integrity. Inflammatory states characterized by elevated G-CSF disrupt this balance, leading to loss of Spi-C^+^ EBIMφs and linking systemic inflammation to defective erythropoiesis and anemia of inflammation. Thus, Spi-C enables macrophages within the EBI niche to adapt to competing metabolic and inflammatory demands, and its dysregulation represents a mechanistic pathway through which inflammatory stress impairs red blood cell production.

Spi-C has also emerged as a critical regulator of inflammation-induced stress erythropoiesis. Stress erythropoiesis is an adaptive, emergency form of red blood cell production that is activated when normal (steady-state) bone marrow erythropoiesis is impaired or insufficient—such as during inflammation, anemia, hypoxia, hemolysis, or infection [34,35,36]. In a murine model of sterile inflammation, Bennett et al. demonstrated that Toll-like receptor signaling promotes erythrocyte phagocytosis by splenic macrophages, leading to increased intracellular heme and induction of Spi-C [37]. Spi-C cooperated with inflammatory signaling pathways to drive macrophage production of cytokines that expand stress erythroid progenitors in the spleen. This mechanism enables compensatory erythropoiesis when steady-state bone marrow erythropoiesis is suppressed by inflammation, positioning Spi-C as a molecular link between erythrophagocytosis, inflammatory sensing, and adaptive red blood cell regeneration.

## 4. Spi-C as a Context-Dependent Modulator of Macrophage Inflammatory Programs

Accumulating evidence positions Spi-C as a critical transcriptional regulator that restrains macrophage inflammatory outputs while coupling inflammatory tone to tissue-specific metabolic cues. Two complementary regulatory paradigms have emerged: (i) heme-dependent Spi-C induction in tissue-resident macrophages, particularly within the intestinal mucosa, and (ii) TLR/NF-κB-driven Spi-C induction in activated macrophages, which is further counter-regulated by interferon signaling. Together, these pathways establish Spi-C as a context-dependent “resolution factor” that calibrates macrophage responses during both homeostasis and inflammation.

### 4.1. Heme-Dependent Spi-C Programs Noninflammatory Intestinal Macrophages

In the intestinal lamina propria, CX_3_CR_1_^high^ macrophages are continuously exposed to microbial ligands yet maintain a hyporesponsive, noninflammatory phenotype essential for mucosal tolerance [38,39,40]. Kayama et al. demonstrated that heme serves as a local tissue-derived signal that induces Spi-C expression in intestinal CX_3_CR_1_^high^ macrophages, thereby enforcing this noninflammatory state. Genetic ablation of Spi-C in myeloid cells resulted in heightened susceptibility to dextran sodium sulfate (DSS)-induced colitis, characterized by exacerbated epithelial damage and expansion of Th17 responses, underscoring a nonredundant role for Spi-C in gut homeostasis [12].

Mechanistically, heme-induced Spi-C selectively suppressed a subset of TLR-inducible proinflammatory genes, including *Il6* and *Il1a*, without broadly inhibiting inflammatory signaling [12]. Rather than functioning as a classical DNA-binding repressor at cytokine promoters, Spi-C directly interacted with IRF5, disrupting the formation of the IRF5–NF-κB p65 transcriptional complex required for induction of these genes. This selective repression preserved macrophage antimicrobial competence while preventing excessive cytokine-driven pathology. Importantly, dietary iron availability modulated Spi-C expression in intestinal macrophages, linking nutritional status and mucosal immunity through a heme–Spi-C axis.

Across tissues, Spi-C operates at the intersection of metabolic and inflammatory signaling, yet its functional outputs are strongly shaped by local microenvironmental context. In the intestine, continuous exposure to microbial products and tonic TLR signaling intersect with heme-dependent regulation to calibrate macrophage inflammatory responsiveness. In contrast, in tissues where inflammatory activation is more episodic, Spi-C regulation may be more tightly linked to transient stress or damage-associated cues. Together, these shared regulatory axes and context-specific inputs determine how Spi-C balances inflammatory restraint with specialized macrophage functions in different anatomical sites.

### 4.2. Toll-like Receptor (TLR)-Induced Spi-C as an Anti-Inflammatory Brake in Activated Macrophages

Beyond the intestine, Spi-C is also induced in macrophages following innate immune activation. Alam et al. showed that TLR signaling triggers Spi-C expression through an NF-κB-dependent, heme-independent pathway, identifying Spi-C as a secondary response gene in activated macrophages [14]. In this context, Spi-C restrains inflammatory cytokine production and promotes resolution-phase functions, including restoration of iron efflux via induction of the iron exporter ferroportin.

Spi-C deficiency resulted in exaggerated inflammatory responses in vivo, including elevated TNF-α production in the serum, following systemic lipopolysaccharide (LPS) challenge [14]. At the transcriptional level, Spi-C limited expression of proinflammatory mediators while selectively promoting genes involved in iron handling, thereby linking inflammatory resolution to metabolic reprogramming [14]. Consistent with these in vivo findings, bone marrow-derived macrophages with higher Spi-C expression exhibited reduced induction of proinflammatory cytokines, accompanied by increased expression of anti-inflammatory mediators [14]. Together, these in vivo and in vitro data indicate that Spi-C functions as a transcriptional modulator of macrophage activation, limiting excessive inflammatory responses while promoting a regulatory, iron-handling phenotype.

### 4.3. Counter-Regulation by Interferon Signaling and Engagement in Sterile Inflammation

Although Spi-C induction dampens inflammatory responses and promotes iron efflux in macrophages, such programs are potentially detrimental during active infection, when sustained inflammatory and antimicrobial functions are required. Indeed, Spi-C is induced relatively late following TLR stimulation, peaking approximately 6–8 h after activation, a timeframe that may coincide with the onset of inflammatory resolution rather than pathogen clearance [14]. This temporal pattern raises the question of how Spi-C expression is restrained in the setting of bona fide infection.

Alam et al. identified interferon signaling—particularly interferon-γ (IFNγ)—as a dominant negative regulator of Spi-C expression. During ongoing infection, IFNγ serves as a key signal of active immune response, alerting macrophages and other immune cells to the presence of pathogens and triggering defense mechanisms [41,42,43]. In bone marrow-derived macrophages, IFNγ potently suppressed LPS-induced Spi-C expression in a STAT1-dependent manner, establishing IFNγ–STAT1 signaling as a direct transcriptional checkpoint limiting Spi-C induction [14]. Type I interferons exhibited a similar but substantially weaker suppressive effect, indicating specificity for IFNγ in this regulatory axis. Importantly, recombinant IFNγ pretreatment in vivo likewise inhibited LPS-induced Spi-C expression in mice lungs, confirming the physiological relevance of this pathway [14].

This suppressive effect extended beyond TLR-induced Spi-C expression. IFNγ blockade increased Spi-C levels in the colon at steady state, consistent with the gut’s tonic exposure to microbial signals that normally promote Spi-C expression. In contrast, lungs—used as a control tissue with comparatively low baseline TLR stimulation—showed no increase in Spi-C following IFNγ neutralization under resting conditions, but exhibited enhanced Spi-C induction upon LPS challenge when IFNγ was blocked [14]. Functionally, the anti-inflammatory effects of IFNγ neutralization were partially Spi-C-dependent, as inflammatory markers remained elevated in Spi-C–deficient mice despite IFNγ blockade, positioning Spi-C downstream of IFNγ signaling in the control of inflammatory magnitude.

Notably, IFNγ also suppressed Spi-C expression in macrophages that constitutively express high levels of Spi-C, independent of TLR signaling [14]. Recombinant IFNγ reduced Spi-C and ferroportin expression in splenic red pulp macrophages both in vivo and ex vivo, demonstrating a direct macrophage-intrinsic effect. Moreover, IFNγ inhibited heme-induced Spi-C expression in bone marrow-derived macrophages and further suppressed already low basal Spi-C levels, indicating that IFNγ acts as a universal repressor of Spi-C regardless of the upstream inductive stimulus.

Together, these findings establish IFNγ–STAT1 signaling as a global inhibitory checkpoint that restrains Spi-C expression across macrophage subsets and activation states. This repression likely serves to preserve inflammatory and antimicrobial functions during infection by preventing premature engagement of Spi-C-dependent anti-inflammatory and iron-export programs. Conversely, relief from IFNγ signaling permits Spi-C induction during inflammatory resolution or in noninfectious settings, allowing macrophages to transition toward tissue-protective and homeostatic states.

### 4.4. Spi-C in Hyperinflammation (Secondary Hemophagocytic Lymphohistiocytosis)

**Figure 2:**
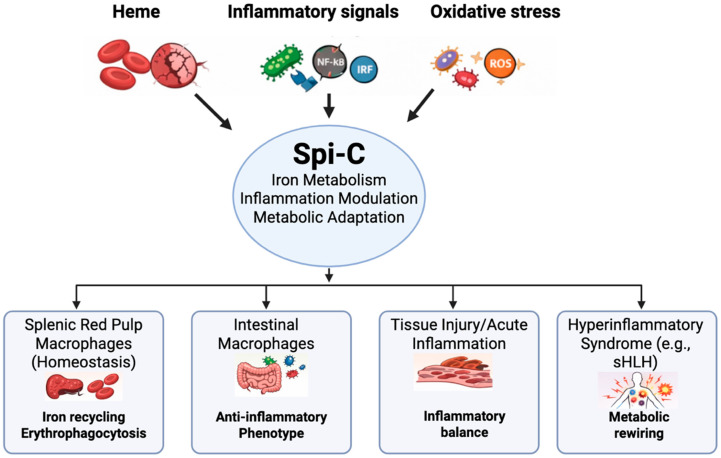
Context-dependent Spi-C regulatory networks across macrophage states. Spi-C integrates heme/iron availability, inflammatory signaling, and oxidative stress cues to shape macrophage transcriptional programs. While supporting erythrocyte clearance and iron recycling in splenic red pulp macrophages under homeostatic conditions, Spi-C also modulates inflammatory tone in intestinal macrophages exposed to tonic microbial signals. During tissue injury or hemolysis, altered heme and redox conditions reshape Spi-C activity, whereas in hyperinflammatory syndromes such as secondary hemophagocytic lymphohistiocytosis, Spi-C-associated programs are upregulated within metabolically rewired macrophages, potentially supporting persistence of pathogenic myeloid activation. Created in BioRender. Alam, Z. (2026) https://BioRender.com/hqw2k3t.

Although Spi-C induction is generally associated with tissue-protective and anti-inflammatory macrophage programs, recent studies demonstrate that Spi-C can also mark a maladaptive macrophage state during severe systemic inflammation (Figure 2). In a model of secondary hemophagocytic lymphohistiocytosis, sequential innate immune stimulation—specifically, viral pattern recognition receptor activation followed by bacterial Toll-like receptor engagement—drives the pathological induction of Spi-C in bone marrow-derived macrophages [44]. Importantly, neither stimulus alone is sufficient to induce disease, highlighting the critical role of signal order and macrophage priming in shaping inflammatory outcomes.

Macrophages exposed to sequential Toll-like receptor stimulation acquire a transcriptional profile that closely resembles that of splenic red pulp macrophages, including the induction of Spi-C and gene networks associated with erythrophagocytosis and iron handling [44]. This red pulp macrophage-like transcriptional signature is observed not only in murine macrophages but also in bone marrow samples from patients with secondary hemophagocytic lymphohistiocytosis, underscoring its clinical relevance. Notably, Spi-C induction in this context occurs ectopically in inflammatory macrophages rather than being restricted to specialized tissue niches, indicating that Spi-C expression can be uncoupled from its physiological developmental context during systemic inflammation [44].

Despite its robust induction, Spi-C is dispensable for disease initiation and mortality in this model, indicating that Spi-C does not function as a primary driver of hyperinflammation. Instead, Spi-C marks a macrophage identity associated with hemophagocytosis, elevated ferritin levels, and inflammatory pathology, positioning it as a transcriptional indicator of a pathological macrophage state rather than a causal effector.

In hyperinflammatory settings, Spi-C activity may not simply be reduced but instead incorporated into a maladaptive inflammatory transcriptional state. While Spi-C can restrain specific pro-inflammatory programs under homeostatic conditions, emerging evidence from models of secondary hemophagocytic lymphohistiocytosis (sHLH) shows upregulation of Spi-C and its associated transcriptional network in highly activated macrophages. In this context of intense Toll-like receptor signaling and metabolic reprogramming toward glycolysis, Spi-C-dependent programs may contribute to sustaining macrophage survival and function within a cytokine-amplifying environment. These findings suggest that Spi-C operates within broader immunometabolic circuits, where its role may shift from maintaining homeostasis to supporting persistence of pathologically activated myeloid cells during severe systemic inflammation.

## 5. Roles of Spi-C Beyond Macrophages

### 5.1. Spi-C in B Cell Fate Decisions

Spi-C is a novel ETS-family transcription factor closely related to PU.1 and Spi-B, but with a distinct DNA-binding and regulatory profile, marking it as a unique regulator within the B lymphoid lineage. Spi-C expression is temporally regulated, emerging predominantly in peripheral, mature B cells while absent in early B cell progenitors, pre-B cells, and terminally differentiated plasma cells [30,45,46]. This restricted expression pattern suggests a role for Spi-C in fine-tuning the transition from immature to mature B cells rather than in early lineage commitment or plasma cell function.

Mechanistically, Spi-C shares considerable homology with PU.1 and Spi-B within its DNA-binding domain, particularly in helices 2 and 3, which mediate direct DNA contacts. Despite these similarities, Spi-C diverges from PU.1 and Spi-B in helix 1, potentially altering protein-protein interactions and thus conferring unique transcriptional regulatory capabilities [47]. Functional studies demonstrate that Spi-C binds canonical PU.1 motifs, including the SP6 κ promoter κY element, and can transactivate reporter constructs in vitro with efficacy comparable to PU.1 [47]. However, unlike PU.1 or Spi-B, Spi-C may also antagonize other Ets factors, as suggested by studies on the related Prf protein, which binds PU.1 motifs in a stage-specific manner and may act as a competitive repressor during B cell development [46].

Spi-C’s regulatory function is further linked to Bach2, a transcription factor critical for B cell fate decisions. By modulating Bach2 expression, Spi-C contributes to the balance between memory B cell and plasma cell differentiation [30]. High Spi-C levels favor the non-secretory, mature B cell state and may restrain premature plasma cell differentiation, whereas downregulation of Spi-C permits full plasma cell maturation. This indicates that Spi-C acts as a temporal checkpoint in the late stages of B cell differentiation, coordinating transcriptional programs with developmental stage-specific cues.

Collectively, these studies position Spi-C as a stage-restricted transcriptional regulator that ensures proper peripheral B cell maturation and function. Its ability to both cooperate with and antagonize related ETS proteins, combined with temporal expression control, highlights its unique contribution to B cell homeostasis and immune readiness.

### 5.2. Spi-C in Stem Cell Metabolism and Epigenetics

Recent studies have uncovered a novel function of Spi-C in embryonic stem cells (ESCs), expanding its role beyond hematopoiesis. Spi-C serves as a marker of ground-state pluripotency, with expression rapidly induced in 2iL-ESCs and in early preimplantation embryos in response to MEK/ERK inhibition [15]. At the molecular level, Spi-C binds to enhancer elements co-occupied by core pluripotency factors, particularly NANOG, stabilizing its chromatin association at genes involved in choline-derived one-carbon (1C) metabolism, including Bhmt, Bhmt2, and Dmgdh [15]. Functional perturbation experiments show that Spi-C activates choline/one-carbon (1C) metabolism, increasing the flux of S-adenosyl methionine (SAM) to S-adenosyl-L-homocysteine (SAH) and modulating epigenetic landscapes. Specifically, Spi-C reduces H3K4me3 and increases H3R17me2a levels, histone marks associated with naïve pluripotency, without affecting global DNA methylation. These epigenetic consequences slow exit from the ground-state pluripotency and confer resistance to environmental perturbations affecting folate metabolism [15]. This observation suggests Spi-C’s role as a metabolic–epigenetic integrator, linking transcriptional regulation, cellular metabolism, and chromatin modifications in ESCs. While outside the macrophage lineage, Spi-C functions in pluripotent stem cells highlight its broader capacity to link metabolic state with epigenetic and transcriptional regulation. These observations reinforce the concept that Spi-C integrates metabolic cues with gene regulatory programs—a principle that may extend to tissue macrophage specialization, where local metabolic environments and inflammatory signals shape lineage-specific transcriptional outcomes.

## 6. Outstanding Questions and Perspectives

Despite growing insight into Spi-C as a transcriptional regulator linking macrophage identity, iron metabolism, and inflammatory control, several critical questions remain. A particularly compelling issue is whether Spi-C regulates susceptibility to ferroptosis. Ferroptosis is an iron-dependent form of regulated cell death characterized by lethal lipid peroxidation and loss of membrane integrity, and mechanistically distinct from apoptosis, necroptosis, and pyroptosis, which are primarily driven by caspase activation or inflammatory pore formation [48,49,50,51]. Ferroptosis has emerged as a key pathogenic mechanism in diverse inflammatory and neurodegenerative diseases, including acute lung injury, sepsis, inflammatory bowel disease, ischemia–reperfusion injury, Alzheimer’s disease, and Parkinson’s disease, where dysregulated iron metabolism and oxidative stress are prominent features [52,53,54,55,56,57,58,59]. Given Spi-C’s central role in erythrophagocytosis, intracellular iron handling, ferroportin-mediated iron export, and metabolic reprogramming, Spi-C is uniquely positioned to influence ferroptotic vulnerability in macrophages and potentially other cell types.

The proposed link between Spi-C and ferroptosis should be viewed as a conceptual framework rather than a demonstrated pathway. Spi-C is a central regulator of macrophage iron handling and heme-responsive transcriptional programs, whereas ferroptosis represents an iron-dependent, lipid peroxidation-driven form of regulated cell death. Although these processes intersect at the level of iron metabolism and redox balance, whether Spi-C-dependent gene networks directly modulate macrophage susceptibility to ferroptotic stress remains to be experimentally defined.

Determining whether Spi-C promotes ferroptosis resistance in iron-rich, homeostatic niches, or instead exacerbates lipid peroxidation and oxidative injury during systemic inflammation, will be essential for understanding macrophage survival and function in disease.

Another important question remains how Spi-C-mediated signaling is regulated across different tissue macrophage populations. Although IRF5–NF-κB cooperation in restraining Spi-C activity has been most clearly characterized in intestinal macrophages, related inflammatory transcriptional circuits likely operate in multiple myeloid populations. In bone marrow-derived macrophages, NF-κB-mediated signaling has been associated with *Spic* expression, potentially downstream of LPS or other inflammatory stimuli; however, the direct upstream mechanisms remain unexplored. In other tissues, such as lungs, the pathways governing Spi-C in inflammatory contexts have not been extensively investigated. Whether the IRF5–NF-κB module or alternative signaling networks control Spi-C expression in these diverse tissue environments remains an open and important question for future studies.

In parallel, it remains unclear how Spi-C cooperates with or competes against PU.1 at a genome-wide level to reshape enhancer landscapes and selectively tune inflammatory versus metabolic gene programs. From a translational perspective, Spi-C’s context-dependent functions raise the possibility of therapeutic targeting in conditions such as hemophagocytic lymphohistiocytosis, colitis, and inflammatory lung disease, while also underscoring the need for precision approaches in respect to tissue- and disease-specific roles. Finally, the diverse actions of Spi-C across macrophages, B cells, and stem cells point to the existence of tissue-specific cofactors and chromatin environments that dictate Spi-C activity. Elucidating these regulatory partnerships will be critical for defining Spi-C as a unifying metabolic–transcriptional node and for harnessing its functions in inflammatory and degenerative disease.

## Data Availability

No new data were created or analyzed in this study. Data sharing is not applicable to this review article.
